# Supplementary material for: Quantifying patient- and hospital-level antimicrobial resistance dynamics in Staphylococcus aureus from routinely collected data
Source: medRxiv. 2023 Feb 16:2023.02.15.23285946. Preprint. [Version 1] doi: 10.1101/2023.02.15.23285946 (PMC9949191; doi:10.1101/2023.02.15.23285946)
Supplement: Supplement 1 [file media-1.pdf]

Quantifying patient- and hospital-level antimicrobial resistance dynamics in *Staphylococcus aureus* from routinely collected data

Quentin Leclerc[1,2,3]*, Alastair Clements[1,3], Helen Dunn[4], James Hatcher[4], Jodi A Lindsay[3], Louis Grandjean[5], Gwenan M Knight[1,2]

[1] Centre for Mathematical Modelling of Infectious Diseases, Department of Infectious Disease Epidemiology, Faculty of Epidemiology & Public Health, London School of Hygiene & Tropical Medicine, UK
[2] Antimicrobial Resistance Centre, London School of Hygiene & Tropical Medicine, UK
[3] Institute for Infection & Immunity, St George's University of London, UK
[4] Great Ormond Street Hospital, UK
[5] Department of Infection, Immunity & Inflammation, Institute of Child Health, University College London, UK

* current address: Epidemiology and Modelling of Bacterial Escape to Antimicrobials, Institut Pasteur, Paris, France (quentin.leclerc@pasteur.fr)

Supplementary material

Supplementary Table 1: Matching information for antibiotics and antibiotic classes.

Antibiotic	Class	Antibiotic	Class
Amikacin	Aminoglycoside	Daptomycin	Lipopeptide
Gentamicin	Aminoglycoside	Azithromycin	Macrolide
Tobramycin	Aminoglycoside	Clarithromycin	Macrolide
Rifaximin	Ansamycin	Erythromycin	Macrolide
Bedaquiline	Bedaquiline	Metronidazole	Metronidazole
Ertapenem	Carbapenem	Mitomycin	Mitomycin
Imipenem	Carbapenem	Aztreonam	Monobactam
Meropenem	Carbapenem	Mupirocin	Mupirocin
Cefalexin	Cephalosporin	Nitrofurantoin	Nitrofuran
Cefiderocol	Cephalosporin	Linezolid	Oxazolidinone
Cefixime	Cephalosporin	Tedizolid	Oxazolidinone
Cefotaxime	Cephalosporin	Amoxicillin	Penicillin
Cefoxitin	Cephalosporin	Ampicillin	Penicillin
Ceftazidime	Cephalosporin	Benzylpenicillin	Penicillin
Ceftolozane	Cephalosporin	Co-Amoxiclav	Penicillin
Ceftriaxone	Cephalosporin	Flucloxacillin	Penicillin
Cefuroxime	Cephalosporin	Phenoxymethylpenicillin	Penicillin
Chloramphenicol	Cephalosporin	Piperacillin	Penicillin
Chlorhexidine	Chlorhexidine	Pivmecillinam	Penicillin
Clofazimine	Clofazimine	Temocillin	Penicillin
Dapsone	Dapsone	Colistin	Polypeptide
Ethambutol	Ethambutol	Protionamide	Protionamide
Ciprofloxacin	Fluoroquinolone	Pyrazinamide	Pyrazinamide
Levofloxacin	Fluoroquinolone	Rifampicin	Rifamycin
Moxifloxacin	Fluoroquinolone	Co-Trimoxazole	Sulfonamide
Ofloxacin	Fluoroquinolone	Sulfadiazine	Sulfonamide
Fosfomycin	Fosfomycin	Trimethoprim	Sulfonamide

Fucidin	Fucidin		Doxycycline	Tetracycline
Teicoplanin	Glycopeptide		Minocycline	Tetracycline
Vancomycin	Glycopeptide		Tetracycline	Tetracycline
Isoniazid	Isoniazid		Tigecycline	Tetracycline
Clindamycin	Lincosamide		Thalidomide	Thalidomide

Supplementary Table 2: Patient characteristics. IQR: interquartile range. LoS: length of stay. Hospital stays shorter than 2 days are not counted in the table and not included in the median LoS estimation.

Year	Number of unique patients	Female patients (%)	Median age (IQR)	"Other Groups" ethnicity (%)	Number of hospital stays	Median LoS (IQR)
**2000**	1103	44.35	4 (1-10)	49.37	2311	5 (3-11)
**2001**	1260	42.49	4 (1-10)	50.25	2770	4 (3-10)
**2002**	1286	43.49	4 (1-10)	46.32	2836	4 (2-10)
**2003**	1223	45.04	5 (1-10)	46.58	2791	4 (2-10)
**2004**	1384	44.03	5 (1-10)	48.52	2875	4 (2-10)
**2005**	1419	43.93	5 (1-10)	47.02	2558	4 (2-10)
**2006**	1451	42.14	5 (1-10)	48.44	2520	4 (2-11)
**2007**	1501	45.4	5 (1-11)	48.72	2381	5 (2-11)
**2008**	1481	45.61	4 (1-10)	49.43	2409	5 (2-10)
**2009**	1435	43.38	4 (1-10)	49.61	2312	5 (2-11)
**2010**	1587	41.51	4 (1-10)	53.39	2413	5 (3-11)
**2011**	1573	44.05	4 (1-10)	49.24	2330	5 (2-11)
**2012**	1478	45.23	4 (1-10)	50.22	2308	5 (3-13)
**2013**	1558	43.29	4 (1-10)	51.80	2387	5 (3-13)
**2014**	1569	44.52	5 (1-11)	52.14	2254	5 (3-12)
**2015**	1490	46.44	5 (1-10)	54.41	2227	6 (3-13)
**2016**	1246	45.86	5 (1-11)	58.42	2222	6 (3-13)
**2017**	1321	45.75	5 (1-11)	57.52	2164	5.5 (3-14)
**2018**	1280	45.06	5 (1-10)	61.40	2265	6 (3-14)
**2019**	1497	44.01	5 (1-10)	57.61	2124	6 (3-14)

**2020**	1337	46.30	5 (1-11)	58.19	1895	6 (3-14)
**2021**	1366	45.61	6 (1-11)	55.54	1720	6 (3-13)

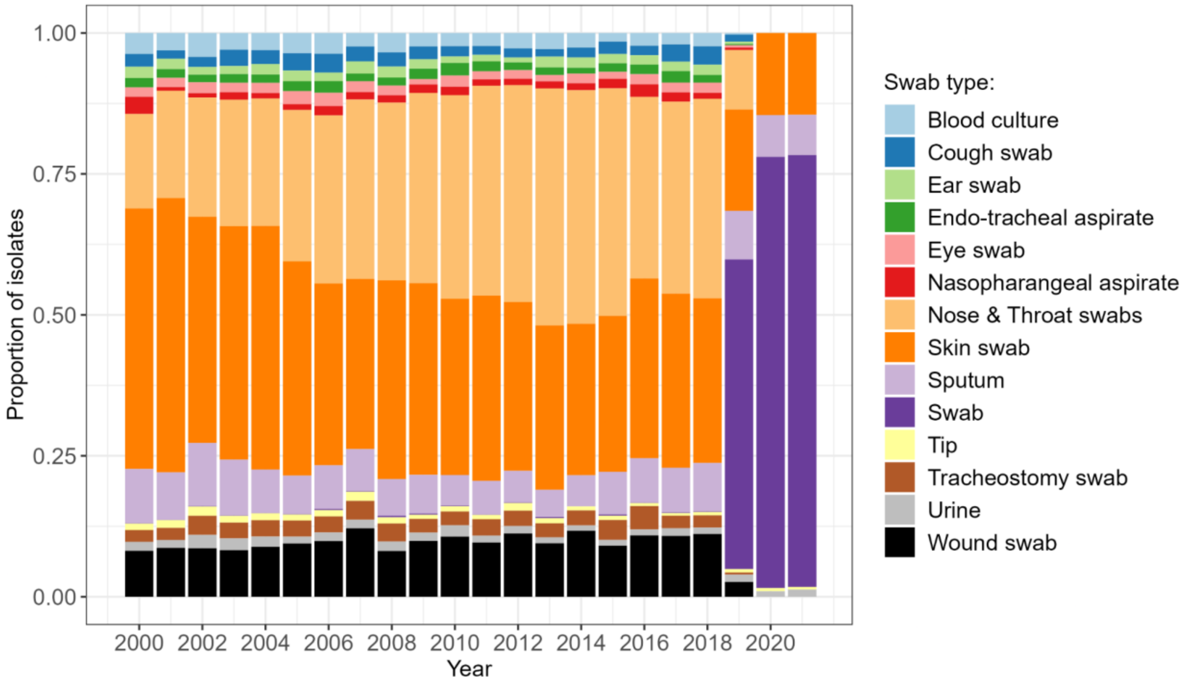

Supplementary Figure 1: Distribution of *S. aureus* isolate sources over time. Only the 14 most common sources across the entire dataset (covering 93% of all isolates) are shown for ease of visualisation.

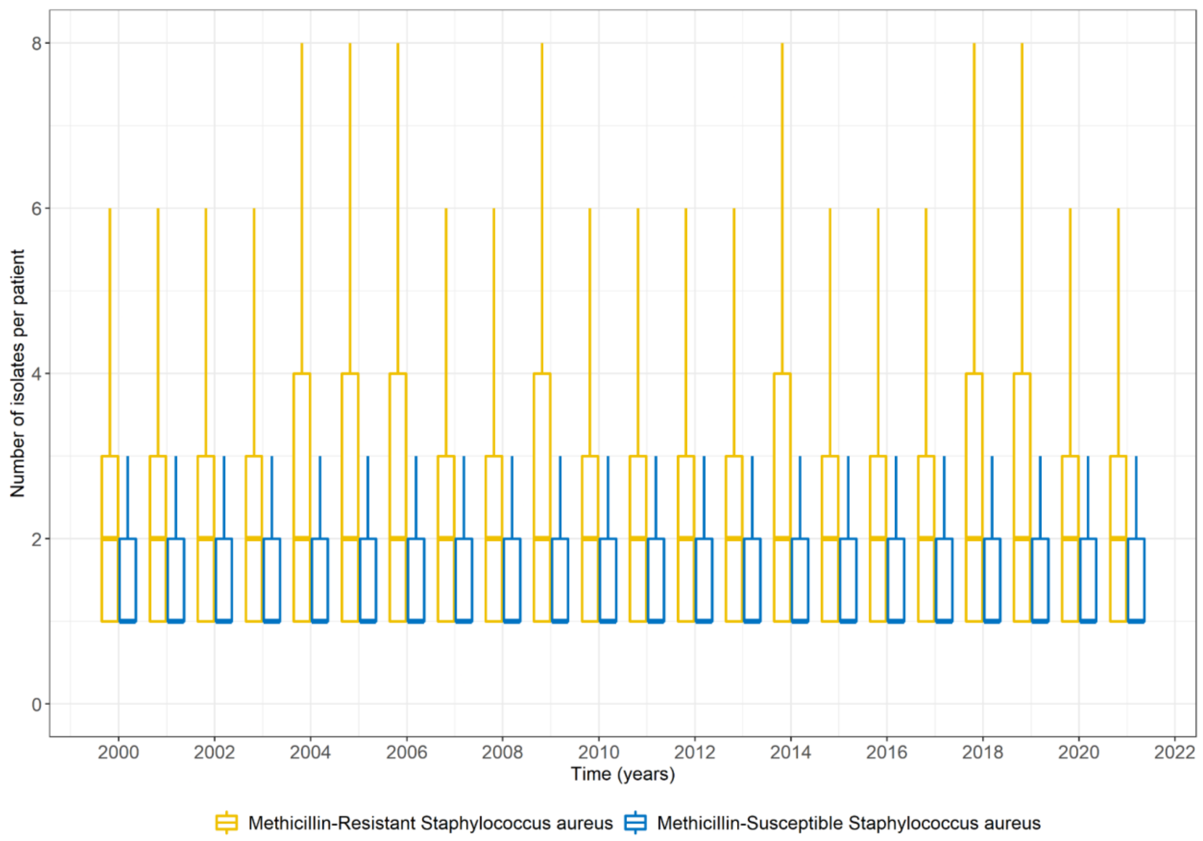

Supplementary Figure 2: Number of MRSA or MSSA isolates per patient per year. Bold lines show the median, box limits show the interquartile range, line limits show the largest value within 1.5 interquartile range above the 75th percentile and smallest value within 1.5 interquartile range below the 25th percentile. Outliers are not shown on the figure.

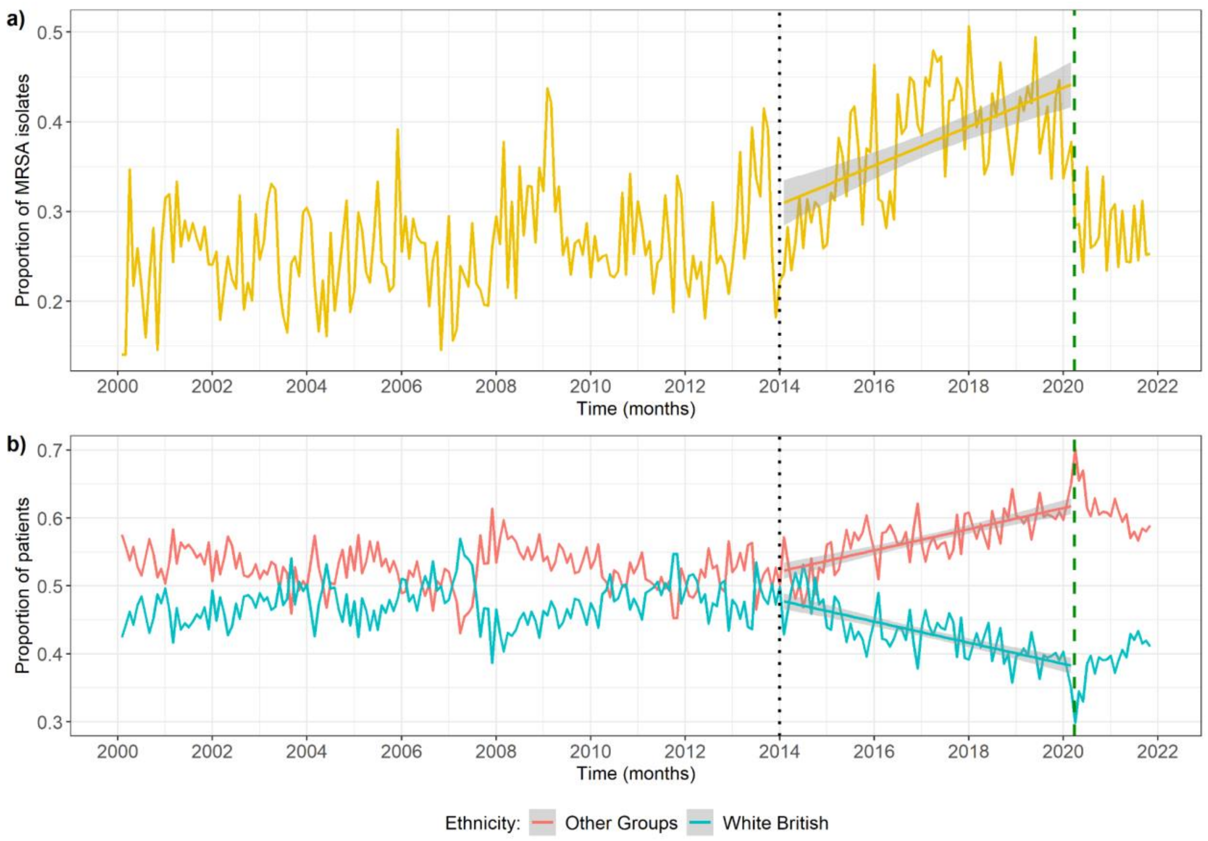

Supplementary Figure 3: Proportion of all *S. aureus* isolates which are meticillin-resistant (a) and ethnicity of patients in our dataset (b). Vertical green dashed line shows the date when lockdown began in the UK during the first wave of the COVID-19 pandemic (29/03/20). Patients with no recorded ethnicity are excluded from the analysis. The interval between the dotted black line (01/01/14) and the green line shows the period where the proportion of MRSA isolates and proportion of patients with an ethnicity of "Other Groups" both increased. The solid lines show the linear regression trendlines between time and these variables.

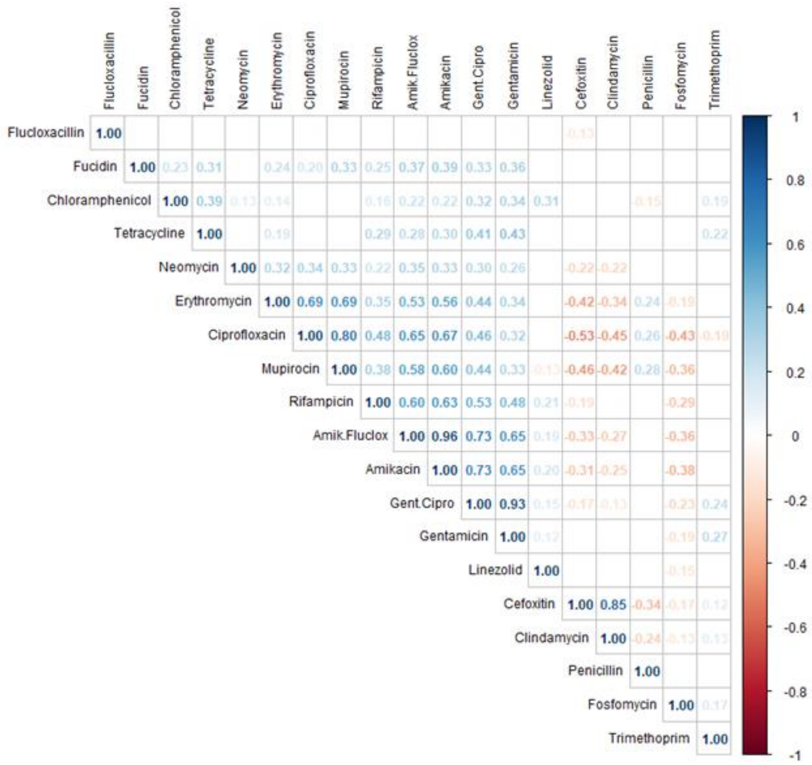

Supplementary Figure 4: Spearman's correlation coefficients between proportions of meticillin-resistant *S. aureus* isolates resistant to different antibiotics over time. Antibiotics included are those with at least 50 susceptibility tests conducted over the entire time period (2000-2021). Only significant coefficients are shown (p value < 0.05).

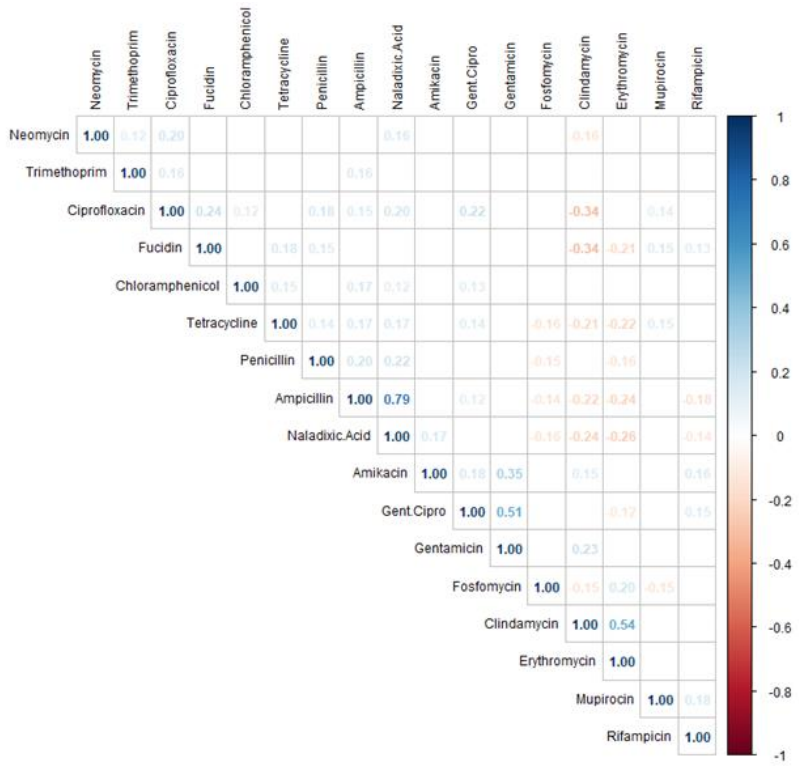

Supplementary Figure 5: Spearman's correlation coefficients between proportions of meticillin-susceptible *S. aureus* isolates resistant to different antibiotics over time. Antibiotics included are those with at least 50 susceptibility tests conducted over the entire time period (2000-2021). Only significant coefficients are shown (p value < 0.05).

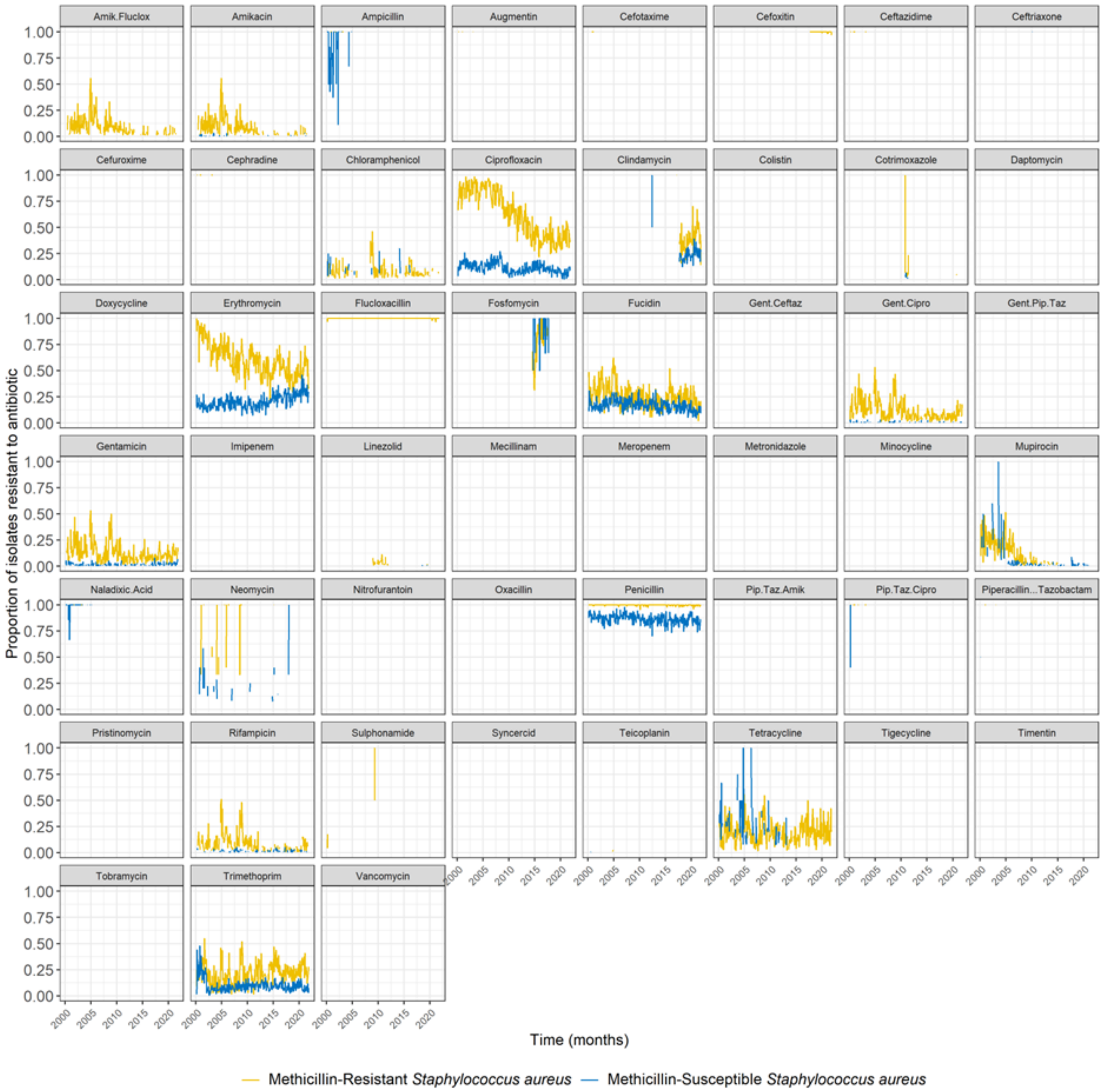

Supplementary Figure 6: Change in proportion of *S. aureus* isolates resistant to antibiotics over time, out of those tested for resistance to the corresponding antibiotic. Amik.Fluclox: joint amikacin and flucloxacillin resistance, Gent.Ceftaz: joint gentamicin and ceftazidime resistance, Gent.Cipro: joint gentamicin and ciprofloxacin resistance, Gent.Pip.Taz: joint gentamicin and piperacillin-tazobactam resistance, Pip.Taz.Amik: joint piperacillin-tazobactam and amikacin resistance, Pip.Taz.Cipro: joint piperacillin-tazobactam and ciprofloxacin resistance. Missing lines indicate that no resistance to the corresponding antibiotic was detected at that time (which may be due to no testing having been conducted at that time).

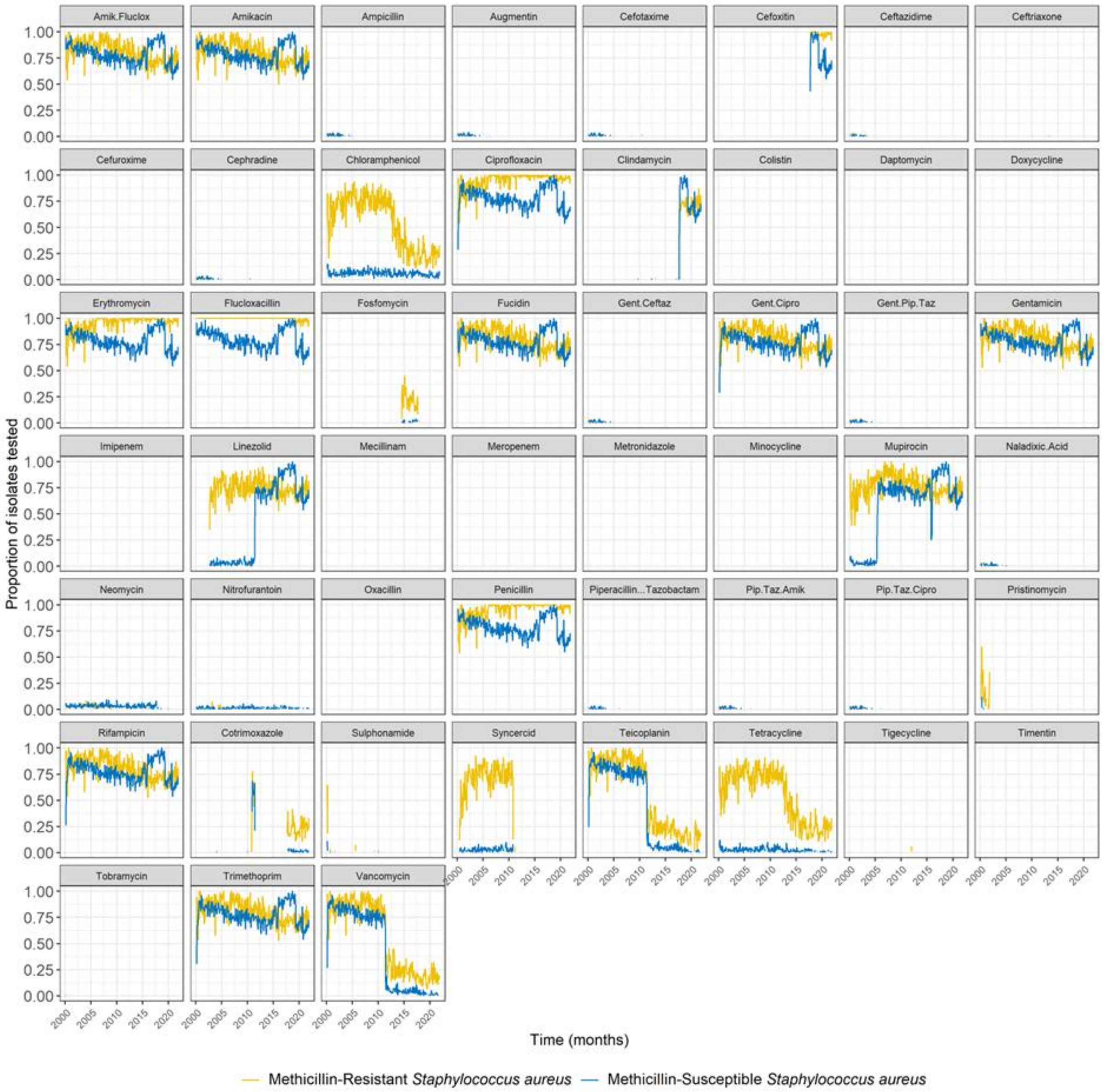

Supplementary Figure 7: Change in proportion of *S. aureus* isolates tested for different antibiotic susceptibilities over time. Amik.Fluclox: joint amikacin and flucloxacillin resistance, Gent.Ceftaz: joint gentamicin and ceftazidime resistance, Gent.Cipro: joint gentamicin and ciprofloxacin resistance, Gent.Pip.Taz: joint gentamicin and piperacillin-tazobactam resistance, Pip.Taz.Amik: joint piperacillin-tazobactam and amikacin resistance, Pip.Taz.Cipro: joint piperacillin-tazobactam and ciprofloxacin resistance. Missing lines indicate that no susceptibility testing for the corresponding antibiotic was conducted at that time.

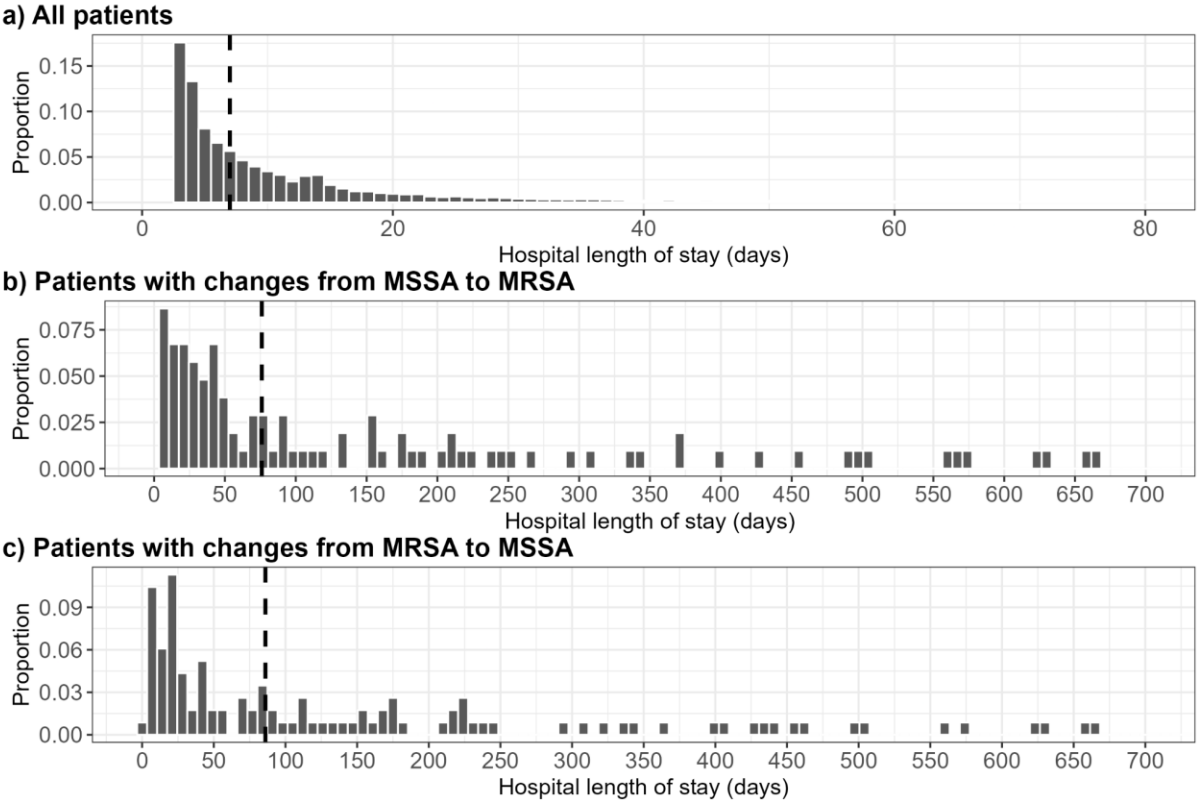

Supplementary Figure 8: Distribution of hospital length of stay (LoS) for patients in our dataset. Some patients may have multiple, separate hospital stays. **a) LoS for all patients.** The dashed line shows the median LoS (7 days). Bin size is 1 day. LoS smaller than 2 days were not included. We excluded 1,342 LoS (3.4%) greater than 80 days from the figure for ease of visualisation. **b) LoS for patients with a change from MSSA to MRSA.** The dashed line shows the median LoS (67 days). Bin size is 7 days. We excluded 4 LoS (3.8%) greater than 700 days from the figure for ease of visualisation. **c) LoS for patients with a change from MRSA to MSSA.** The dashed line shows the median LoS (86 days). Bin size is 7 days. We excluded 5 LoS (4.3%) greater than 700 days from the figure for ease of visualisation.

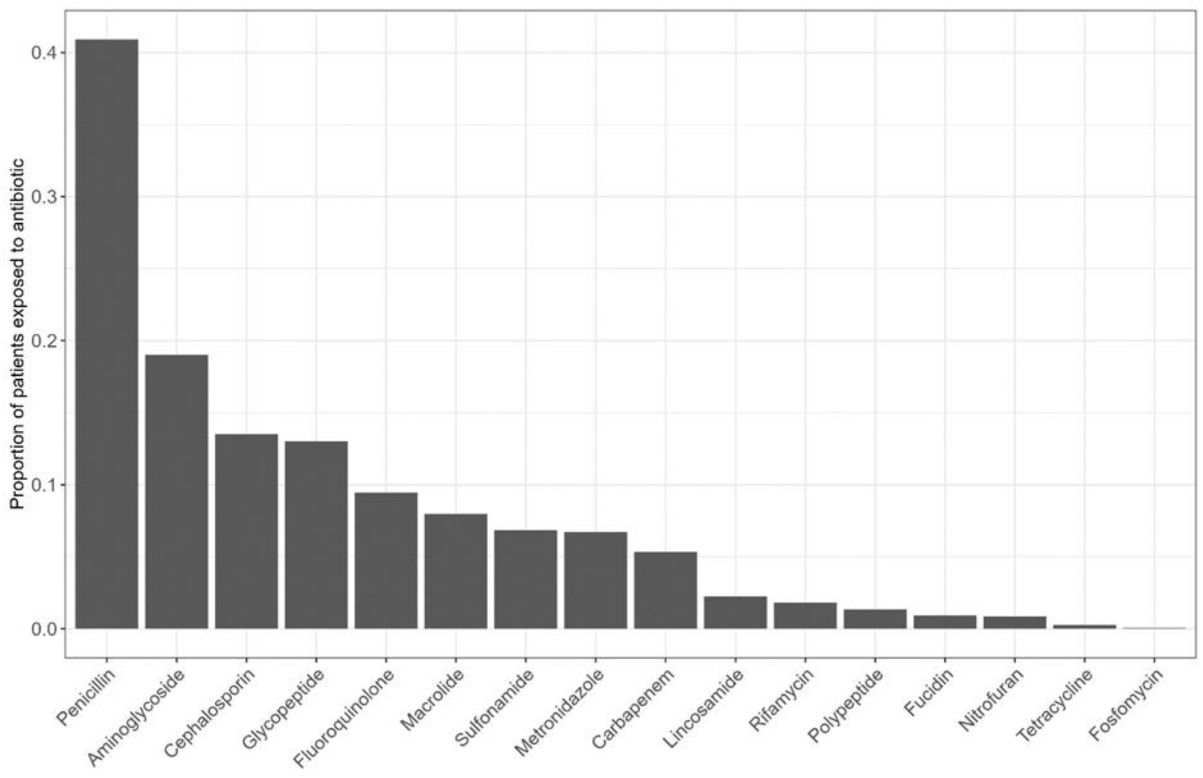

Supplementary Figure 9: Proportion of all patients with a positive *S. aureus* swab exposed to antibiotics of different classes. Patients may have been exposed to more than one antibiotic.
